# Dynamic changes and functions of macrophages and M1/M2 subpopulations during ulcerative colitis-associated carcinogenesis in an AOM/DSS mouse model

**DOI:** 10.3892/mmr.2014.3018

**Published:** 2014-11-28

**Authors:** WEI WANG, XIAYU LI, DANWEI ZHENG, DECAI ZHANG, XIAOQING PENG, XUEMEI ZHANG, FEIYAN AI, XIAOYAN WANG, JIAN MA, WEI XIONG, GUIYUAN LI, YANHONG ZHOU, SHOURONG SHEN

**Affiliations:** 1 Department of Gastroenterology, The Third Xiangya Hospital of Central South University, Changsha, Hunan 410013, P.R. China; 2 Hunan Key Laboratory of Nonresolving Inflammation and Cancer, Disease Genome Research Center, Changsha, Hunan 410078, P.R. China; 3 Department of Gastroenterology, The Jiangmen Central Hospital, Jiangmen, Guangdong 529030, P.R. China; 4 Key Laboratory of Carcinogenesis, Ministry of Education, Cancer Research Institute, Changsha, Hunan 410078, P.R. China; 5 Key Laboratory of Carcinogenesis and Cancer Invasion, Ministry of Education, Cancer Research Institute, Changsha, Hunan 410078, P.R. China

**Keywords:** macrophages, dynamic changes, ulcerative colitis, carcinogenesis

## Abstract

The high risk of developing colorectal carcinoma (CRC), from ulcerative colitis (UC), is well known. Macrophages are widely distributed immune cells that have an indispensable role in UC, as well as in CRC. However, little is currently known about the dynamic changes that occur in macrophage and M1/M2 macrophage subpopulations, during UC-associated carcinogenesis. The aim of the present study was to investigate the alteration of colorectal macrophages and M1/M2 macrophage subpopulations during UC-associated carcinogenesis. Both expression level alterations and functional changes were determined during UC-associated carcinogenesis in an azoxymethane/dextran sodium sulfate-induced chemically colitis-associated carcinoma mouse model of Crj:CD-1 (ICR) mice. Notable evidence from immunohistochemistry, flow cytometry, cytokine detection, and gene expression analyses demonstrated that M2 macrophages have a critical role in CRC initiation, promotion, and metastasis. M2 macrophages are associated with unbalanced pro-inflammatory and anti-inflammatory axes and aberrant enhancement of migration/invasion-associated factors. Functional changes, similar to M2 polarized macrophages, were shown to occur in the M1 macrophages, without phenotypical changes, during the development of carcinoma and metastasis. The results of the present study suggest that M2 macrophages have a pro-tumor role during UC-associated carcinogenesis. Furthermore, similar functional changes occurred in the M1 macrophages, without polarization alterations, during carcinogenesis and metastasis.

## Introduction

Colorectal carcinoma (CRC) is one of the most common malignancies worldwide and the most common cause of cancer-associated mortality in China (1). Ulcerative colitis (UC) is an idiopathic colorectal inflammatory disorder that has a high risk of developing into CRC if not effectively treated, at which point it cannot be resolved (2–5). As an adenoma-carcinoma sequence, UC-CRC undergoes a series of inflammatory processes and dysplasia in order to develop into carcinoma; this is known as the ‘inflammation-cancer link’ (6). The development of UC-CRC occurs through a multi-factorial and multi-step evolution of molecular events, that includes the activities of nuclear factor (NF)-κB and signal transducer and activator of transcription-3 signaling pathways (7–10). However, numerous key questions regarding the molecular mechanisms and the involvement of subpopulations of immune cells, remain unanswered, particularly regarding the microenvironment during UC-CRC carcinogenesis.

UC is an autoimmune disease that affects numerous organs, as well as the digestive system, and the development of UC-CRC is associated with immune system malfunction (11). Macrophages have an indispensable role in inflammation and carcinoma. Activated macrophages can mount specific functions, which may be either pro-inflammatory or anti-inflammatory, thereby contributing to local tissue destruction, or regeneration and wound healing. These mechanisms are dependent on the polarized phenotypes that the macrophages acquire, within that particular microenvironment (12). Polarized macrophages are mainly classified into two groups: Classically activated macrophages, M1; and alternatively activated macrophages, M2 (13). M1 macrophages are characterized by the secretion of pro-inflammatory cytokines, including interleukin (IL)-1β, IL-6, IL-12, and tumor necrosis factor (TNF)-α. Conversely, M2 cells induce a weak immune response and reduce pro-inflammatory responses (14). However, the mechanisms responsible for the changes in M1/M2 polarization, and their functions in UC-CRC carcinogenesis, have yet to be elucidated.

Previous studies have shown that macrophage infiltration is greater in patients with UC, as well as CRC, as compared with healthy patients (15–17). However, little is currently known about the dynamic and sequential changes that occur in macrophages during the transition from UC to CRC (18–21). In the present study, the variable roles of colorectal macrophages and subpopulations were investigated in the changing microenvironment during UC-CRC carcinogenesis. M1/M2 macrophages were purified and sorted from a chemically-induced mouse model, in order to evaluate the dynamic changes of their expression and functions during the various phases of the inflammation-cancer process.

## Materials and methods

### Animals and experimental procedure

All of the procedures in the present study involving mice were performed in accordance with institutional guidelines and the Ethics Committee Concerning the Care and Use of Experimental Animals provided by the Institutional Review Board of The Third Xiangya Hospital (Changsha, China). An azoxymethane (AOM) and dextran sodium sulfate (DSS)-induced colitis-associated carcinoma mouse model was used in the present study, which has been described by previous methods (22–24). A total of 96 six-week-old male Crj:CD-1 (ICR) mice, purchased from Hunan Slack King of Laboratory Animals Co., Ltd., (Changsha, China) were randomized into either the AOM/DSS group or the control group. The Disease Activity Index (DAI) was used to evaluate disease development, by scoring body weight loss, stool characterization and hematochezia, as previously described (25). The two groups of mice were randomly, sequentially sacrificed by exsanguination at the end of week 0 (before treatment), and at weeks 2, 4, 7, 10, 16, 24 and 56 post-treatment. The mice were anesthetized with a cotton ball dipped in ~0.5–1 ml ethyl ether in a sealed chamber, and were sacrificed by exsanguination, through eye enucleation. Spontaneous activity and the corneal reflex disappeared and no significant contraction was observed in the limb muscles on stimulation.

### Histopathology and immunohistochemistry (IHC)

The mice were anaesthetized and sacrificed by exsanguination, as detailed above. The colon was then incised longitudinally along the main axis and washed twice with 1X phosphate-buffered saline (PBS). Following careful macroscopic inspection, half of the distal colon and rectum was cut longitudinally, fixed with 4% paraformaldehyde, embedded in paraffin, and subsequently cut into 5 μm sections. The sections were then stained with hematoxylin and eosin, and assessed by two investigators in a blind manner to provide a consensus on staining patterns.

Immunohistochemical staining was performed using a rabbit anti-mouse CD68 (cat.no BA3638; 1:100 dilution; Boster Biological Technology, Inc., Wuhan, China) primary antibody, at 4°C overnight. The sections were then incubated with biotin-conjugated goat anti-rabbit secondary antibody (cat.no. BA1003; 1:2,000 dilution; Boster Biological Technology, Inc.). A 3, 3′-diaminobenzidine (DAB; Boster Biological Technology, Inc.) kit was used for staining, at a 1:20 dilution. Omission of the primary antibody was used as a negative control. The images were captured using a BX51 microscope (Olympus America Inc., Center Valley, PA, USA).

### Macrophage isolation and sorting by fluorescence-activated cell sorting (FACS)

Single-cell suspensions from the colonic tissue samples were generated. The colonic tissue samples were minced into 1 mm^3^ pieces using opthalmic scissors and digested in serum-free Dulbecco’s modified Eagle’s medium (DMEM), supplemented with 1 mg/ml collagenase IV (Invitrogen Life Technologies, Carlsbad, CA, USA), 1% hyaluronidase (Sigma-Aldrich, St Louis, MO, USA), and 0.25% DNase I (Merck Millipore, Darmstadt, Germany). The samples were incubated at 37°C for enzyme digestion, with oscillation every 10–15 min. The normal colon samples were digested for 2–2.5 h and the carcinoma samples were digested for 1–1.5 h, until the tissues were dispersed into cell clusters or single cells. The samples were then passed through a 70 μm cell strainer (BD Biosciences, Franklin Lakes, NJ, USA), and the resulting cell suspension was centrifuged at 500 g for 5 min, and subsequently resuspended in 1X PBS.

The single-cell suspensions were incubated, at 4°C for 30 min, with the following rat anti-mouse antibodies: APC/Cy7-conjugated F4/80 (clone CI:A3–1), APC-conjugated CD68 (clone, FA-11), PerCP/Cy5.5-conjugated CD11b (clone, M1/70), phycoerythrin-conjugated CD16/32 (clone 93) and fluorescein isothiocyanate (FITC)-conjugated CD206 (clone, C068C2). FITC-conjugated rat anti-mouse immunoglobulin G was used as a negative control (clone poly4060). All of the antibodies were purchased from Biolegend (San Diego, CA, USA), and used according to the manufacturer’s instructions. The cells were washed twice with stroke-physiological saline solution of mice (0.75%) and then sorted and assessed by FACS, using a MoFlo^TM^ XDP High-Performance Cell Sorter (Beckman Coulter, Brea, CA, USA). The data were acquired and analyzed using Summit v5.2 software (Beckman Coulter).

### Purified cell cultures and ELISA

Following cell sorting by FACS, the purified cells were cultured for 24 h, at 37°C in a 5% CO_2_ humidified atmosphere, in DMEM (Life Technologies, Grand Island, NY, USA) supplemented with 100 U/ml penicillin, 100 μg/ml streptomycin, and 10% fetal bovine serum (Life Technologies). The cells were centrifuged at 700 g for 10 min and aliquots of the supernatant were frozen at −80°C, until further use. Commercially available ELISA kits for TNF-α, IL-12, and IL-10 were purchased from R&D Systems, Inc. (Minneapolis, MN, USA), and were used according to the manufacturer’s instructions. The results of the ELISA were examined using the Paradigm^TM^ Detection Platform (Beckman Coulter).

### RNA extraction and quantitative polymerase chain reaction (qPCR)

Sorted colorectal M1/M2 macrophages were collected following a 24 h culture. Total RNA was extracted using the RNeasy Micro kit (Qiagen, Hilden, Germany) and cDNA synthesis was conducted using the SuperScript^TM^ III First-Strand Synthesis system (Invitrogen Life Technologies), according to the manufacturer’s instructions.

A qPCR analysis was performed using the GoTaq qPCR Master Mix (Promega Corp., Madison, WI, USA). The primers used were *GAPDH, CXCR4, GCSF, GMCSF, ICAM-1, IL-1β, IL-6, IL-10, IL-12, TGFβ, TNFα,* and *VEGF,* the sequences for which are shown in Table I. The qPCR was performed using a Bio-Rad CFK96^TM^ Real-Time System (Bio-Rad Laboratories, Inc., Hercules, CA, USA). The cycle conditions were as follows: 2 mins at 95°C, 15 sec at 95°C and 1 min at 60°C for 59 cycles, followed by 10 sec at 95°C with a melt curve of 65–95°C and increments of 0.5°C. The data were analyzed using Bio-Rad CFK Manager 2.0 software. The relative gene expression levels were quantified based on the cycle threshold value, and normalized to the housekeeping gene *GAPDH*.

### Statistical analyses

All of the statistical analyses were performed using SPSS version 17.0 software (SPSS Inc., Chicago, IL, USA). The distributions of continuous variables were described by means and standard errors. Statistical significance was determined using the Independent Samples t-test. A P<0.05 was considered to indicate a statistically significant difference.

## Results

### Establishment of the AOM/DSS mouse model showing the sequence of inflammation, inflammatory hyperplasia, dysplasia, and carcinoma to metastasis transition

**Figure 1:**
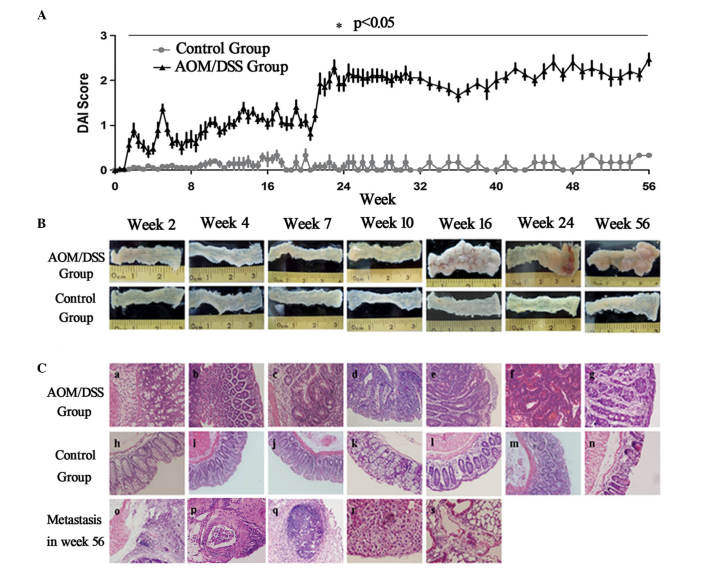
Establishment of inflammation-carcinoma metastasis sequence using the azoxymethane/sextran sodium sulfate (AOM/DSS) mouse model. (A) The Disease Activity Index (DAI) of the AOM/DSS group was higher, as compared with the control group. DAI = (weight loss score + stool characteristic score + hematochezia score)/3, ^*^P<0.05. (B) Morphological observation of the distal colon and rectum cut longitudinally. Colonic mucosa with erosions and ulcers was observed in weeks 2, 4, and 7. Neoplasms were observed from week 10–56 in the AOM/DSS mice, as compared with the control mice. (C) Histopathology of colonic mucosa by hematoxylin and eosin staining (magnification, ×200) In the AOM/DSS group, the colonic mucosa was examined for (a) inflammation at week 2, (b) inflammatory hyperplasia at week 4 and (c) dysplasia at week 7. (d-g) Carcinoma and (o and p) peritoneal metastasis through the intestinal wall was observed at week 56. No metastasis was observed in the (q-s) celiac lymph node, liver, or lung. (h-k) Normal mucosa and (l-n) mild inflammation were observed in the control group mice.

Morphological and pathological analyses of the colonic mucosa revealed a sequential process of inflammation by week 2, inflammatory hyperplasia by week 4, dysplasia by week 7, and carcinoma at weeks 10, 16, 24, and 56. Furthermore, peritoneal metastasis was observed by week 56 in the mice of the AOM/DSS group. Conversely, the control mice exhibited normal mucosa between weeks 2 and 10, and mild inflammation between weeks 16 and 56 (Fig. 1). The morphological and pathological analyses showed that the progression observed was concordant with the UC-CRC process, including metastasis. These results verify the successful establishment of the UC-CRC carcinogenesis transition *in vivo,* using an AOM/DSS mouse model that mimicked the development pattern observed in humans.

### Distribution of colorectal macrophages and M1/M2 subpopulations, during UC-CRC carcinogenesis

**Figure 2:**
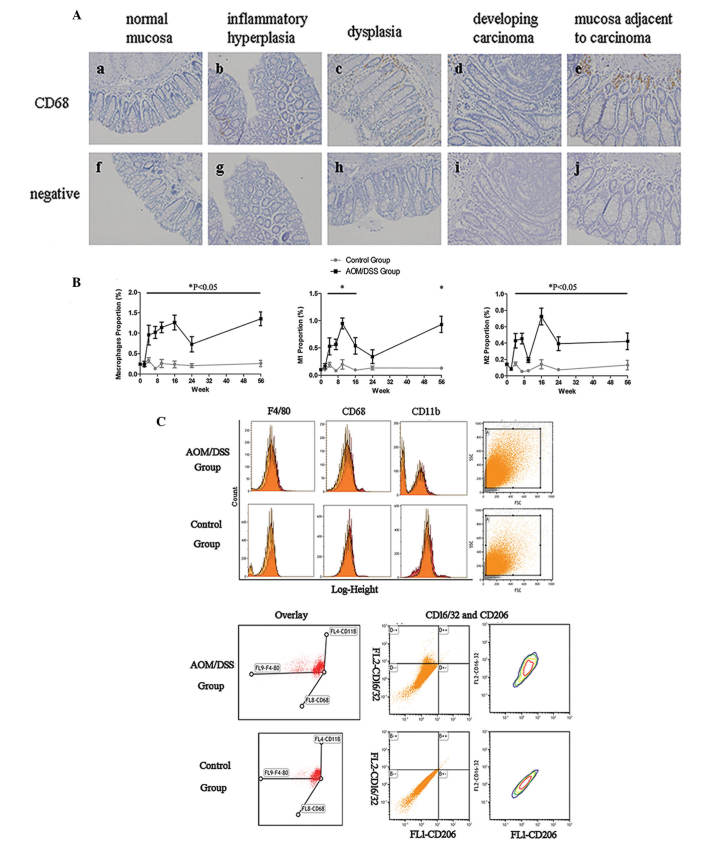
Expression pattern of macrophages and M1/M2 macrophage subpopulations in colonic tissue during ulcerative colitis-colorectal carcinoma (UC-CRC) carcinogenesis. (A) The expression pattern of macrophages was assessed by immunohistochemistry using an anti-CD68 antibody. CD68 was localized to the cell membranes. Hematoxylin was used to stain the nuclei. The expression of CD68^+^ cells increased sequentially from (a) normal mucosa to (b) inflammatory hyperplasia, (c) dysplasia, and (d) carcinoma. (e) The CD68^+^ cells were also observed in the mucosa adjacent to carcinoma tissue. All of the samples were compared to the (f-j) negative control. (B) Colorectal macrophages were evaluated and purified by fluorescence-activated cell sorting, using the indicated surface markers. Further sorting generated the M1 and M2 macrophage subpopulations. ^*^P<0.05. (C) Flow cytometric description of colorectal macrophages and M1/M2 macrophage subpopulations isolated from azoxymethane/sextran sodium sulfate (AOM/DSS) mice at week 4 post-induction. Single-parameter histograms show the expression of macrophage markers F4/80, CD68, or CD11b (red), and negative control (orange), for the two groups. A three dimensional coordinate system shows F4/80^+^CD68^+^CD11b^+^ cells by x, y, and z axes. Two-parameter histograms show F4/80^+^CD68^+^CD11b^+^ cells stained with anti-CD16/32 in the FL2-phycoerythrin channel and anti-CD206 in the FL1-fluorescein isothiocyanate channel. CD16/32 and CD206 were markers for M1 and M2 macrophages, respectively, based on F4/80, CD68, and CD11b staining. All of the samples were compared to the immunoglobulin G isotype control.

To investigate the expression pattern of macrophages in the colonic mucosa during the UC-CRC sequential process, IHC was performed using an antibody specifically targeting the macrophage-specific marker CD68. The percentage of CD68^+^ macrophages increased sequentially from the normal mucosa to inflammatory hyperplasia, dysplasia, and carcinoma transition in the mice of the AOM/DSS group. In addition, a greater number of CD68^+^ cells were observed in the mucosa adjacent to the carcinoma, as compared with the carcinoma tissue itself (Fig. 2A).

To further evaluate the changes in the presence of macrophages and the M1/M2 macrophage subpopulations in the colonic tissue during UC-CRC transition, M1 and M2 macrophages were examined using antibodies targeting distinct cellular markers. CD68, F4/80, and CD11b are widely recognized as surface markers of macrophages in mice. Colorectal macrophages were initially identified in the AOM/DSS mice, by FACS, at week 2 post-induction, and continued to week 10 post-induction, which was the time at which the carcinoma tissue first appeared. The highest macrophage percentage was observed in inflammatory hyperplasia at week 4, which was concordant with the results obtained from the CD68^+^ IHC staining. The percentage of M1 macrophages present in the colon mucosa was similar to that of the total number of macrophages present during this process, but exhibited a marked increase during the transition from dysplasia to carcinoma, between weeks 7 and 10. Conversely, the percentage of M2 macrophages remained constant during this process. The mice in the control group maintained low levels of both M1 and M2 macrophages in the colonic tissue, during the entire experimental period (Fig. 2B and C).

The expression pattern of M1 and M2 macrophages changed as the carcinoma progressed to peritoneal metastasis, from week 10 post-induction. A decrease in the number of M1 and M2 macrophages was observed as the carcinoma progressed *in situ* from week 16–24 post-induction, and an earlier decrease in the number of cells was observed for the M1 subpopulation after week 10, as compared with the M2 subpopulation, which markedly increased between weeks 10 and 16. This increase was followed by an immediate decrease between weeks 16 and 24, suggesting that the expression of M2 macrophages is most likely correlated with the early stages of carcinoma development. As the carcinoma invaded through the colonic mucosa, resulting in peritoneal metastasis, the total number of macrophages markedly increased between weeks 24 and 56. This was similar to the M1 macrophage subpopulation and most likely associated with carcinomatous peritonitis, as a result of the metastasis. Furthermore, a moderate increase in the number of M2 macrophages was maintained during this process. These data suggest that marked changes occur in the M1 and M2 macrophage subpopulations during UC-CRC carcinogenesis transition, particularly during inflammatory hyperplasia. These results indicate that this period during UC-CRC may be a key event in the progression of inflammation to carcinoma.

### Functional changes in the M1/M2 subpopulations during the UC-CRC process

**Figure 3:**
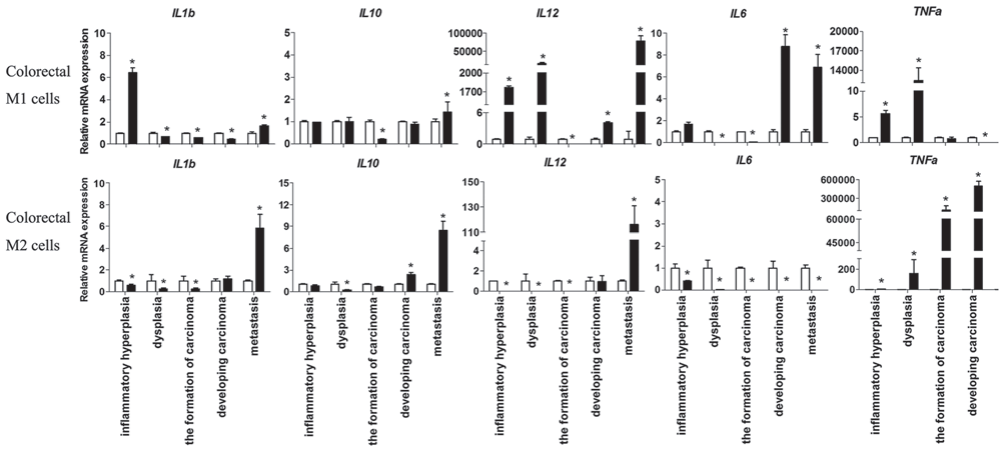
Aberrant expression levels of pro- and anti-inflammatory cytokines in M1/M2 macrophages, during the transition from ulcerative colitis to colorectal carcinoma metastasis. Colorectal M1/M2 macrophage cells were collected, following a 24 h culture, and assessed for mRNA expression levels of pro-and anti-inflammatory cytokines by quantitative polymerase chain reaction. Representative data from at least three independent experiments are shown. ^*^P<0.05. White bars, control group; black bars, azoxymethane/dextran sodium sulfate group; IL, interleukin; TNF-α, tumor necrosis factor-α.

The results of the present study indicate that the inflammatory hyperplasia step, during the UC-CRC process, may be a key point where marked changes occur in the expression patterns of total macrophages, and M1 and M2 subpopulations. These changes are then followed by dysplasia, carcinoma, and metastasis. To evaluate the roles of M1/M2 macrophages in the pro- and anti-inflammatory balance of the tumor microenvironment during this process, the mRNA expression levels of various secreted cytokines, including IL-1β, IL-6, IL-10, IL-12, and TNF-α, were determined by qPCR. Pro-inflammatory cytokines were expressed by colorectal M1 macrophages during inflammatory hyperplasia (IL-1β, IL-6) and dysplasia (IL-12, TNF-α), thus indicating that MI macrophages have a positive role during the process of carcinoma. In addition, high expression levels of the pro-inflammatory factors IL-1β, IL-6, and IL-12, as well as the anti-inflammatory cytokine IL-10, were detected during metastasis. The suppression of anti-inflammatory IL-10 was observed in the colorectal M2 macrophages, which occurred concomitantly with low expression levels of pro-inflammatory cytokines, prior to carcinoma formation. Conversely, high expression levels of pro- and anti-inflammatory cytokines were detected during metastasis (Fig. 3).

**Figure 4:**
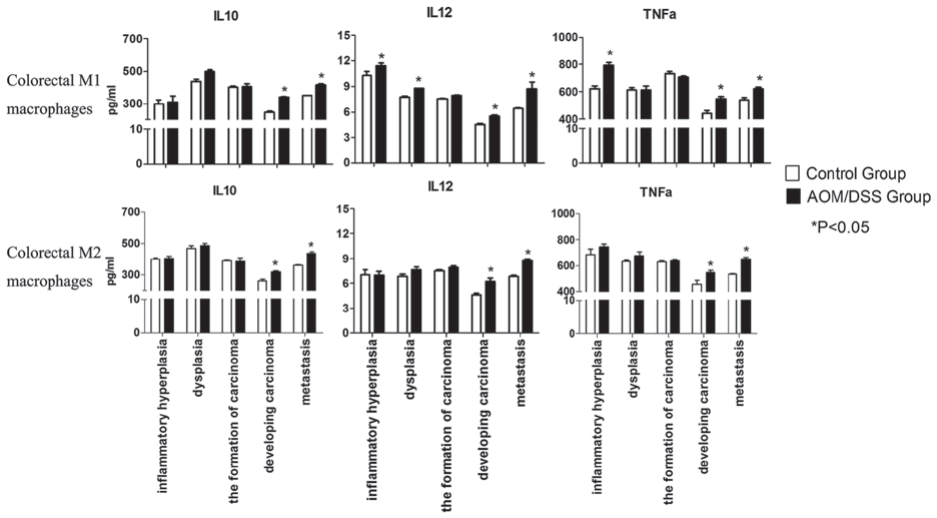
Aberrant expression levels of pro-inflammatory and anti-inflammatory cytokines in M1/M2 macrophages during the transition from ulcerative colitis to colorectal carcinoma metastasis. Colorectal M1/M2 macrophages were cultured for 24 h at 37°C in an atmosphere containing 5% CO_2_, and the culture supernatant was collected to determine the secreted cytokine levels, by ELISA. Representative data from at least three independent experiments are shown. ^*^P<0.05. White bars, control group; black bars, AOM/DSS group; IL, interleukin; TNFa, tumor necrosis factor-α; AOM/DSS, azoxymethane/dextran sodium sulfate.

To investigate further, ELISAs were performed to measure the release of cytokines from the purified M1/M2 macrophages, following a 24 h *in vitro* culture. Concordant with the mRNA expression data, M1-induced IL-12 and TNF-α secretion was markedly upregulated in the inflammatory hyperplasia and metastasis colonic samples from the AOM/DSS mice. Furthermore, there was a concomitant release of IL-10. The suppression of M2-induced IL-10, IL-12, and TNF-α secretion was observed during the transition from inflammatory hyperplasia to carcinoma, but these cytokines were again expressed at high levels during metastasis, which was concordant with the mRNA expression data (Figs. 3 and 4).

**Figure 5:**
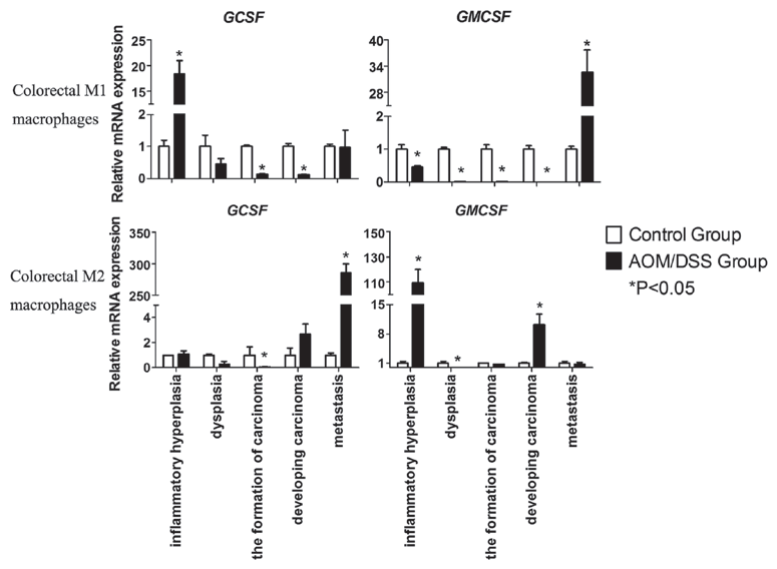
Granulocyte colony-stimulating factor (G-CSF) and granulocyte macrophage colony-stimulating factor (GM-CSF) expression levels in colorectal M1/M2 macrophages, as evaluated by quantitative polymerase chain reaction. Numerous factors are involved in the recruitment and migration of M1/M2 macrophages during ulcerative colitis-colorectal carcinoma carcinogenesis. Representative data from at least three independent experiments are shown. ^*^P<0.05. White bars, control group; black bars, AOM/DSS group; AOM/DSS, azoxymethane/dextran sodium sulfate.

To determine the recruitment and migration of macrophages in CRC and peritoneal metastasis through autocrine regulation, granulocyte colony-stimulating factor (G-CSF) and granulocyte macrophage colony-stimulating factor (GM-CSF) expression levels were determined. G-CSF expression levels were markedly increased in the M1 macrophages from the inflammatory hyperplasia samples and M2 macrophages from the metastasis samples (Fig. 5). Notably, the expression levels of GM-CSF were high in the M2 macrophages isolated from the inflammatory hyperplasia samples and the M1 macrophages from the metastasis samples, as compared with the other stages of the UC-CRC transition (Fig. 5). These results were concordant with the flow cytometric data.

**Figure 6:**
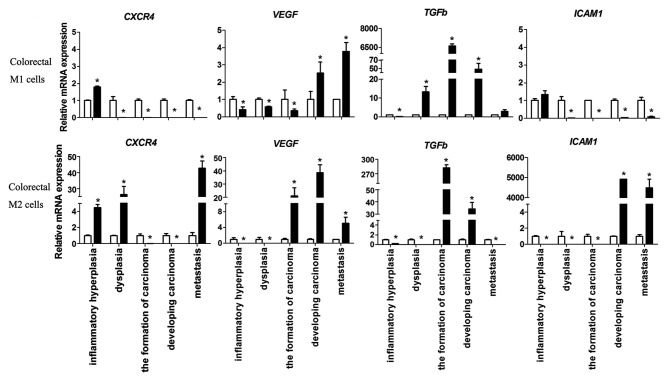
Expression levels of metastasis-associated factors (as shown) in colorectal M1/M2 macrophages, as evaluated by quantitative polymerase chain reaction. Numerous factors are involved in the recruitment and migration of M1/M2 macrophages duringulcerative colitis-colorectal carcinoma carcinogenesis. Representative data from at least three independent experiments are shown. ^*^P<0.05. White bars, control group; black bars, azoxymethane/dextran sodium sulfate group; CXCR4, C-X-C chemokine receptor type 4; VEGF, vascular endothelial growth factor; TGFb, transforming growth factor-β; ICAM1, intracellular adhesion molecule-1.

C-X-C chemokine receptor type 4 (CXCR4) expression levels were elevated in the M2 macrophages isolated from dysplastic and metastatic tissue samples from the AOM/DSS mice, as well as in the M1 macrophages from the inflammatory hyperplasia samples. Furthermore, vascular endothelial growth factor (VEGF) expression levels were markedly elevated in the M1 and M2 macrophages isolated from the carcinoma tissues (Fig. 6). These results indicate that coordinated CXCR4 and VEGF enhancement in colorectal macrophages may contribute to invasion and metastasis of carcinoma, especially in the M1 macrophages during the later stages of the UC-CRC transition. Transforming growth factor (TGF)-β expression levels were markedly elevated in the colorectal macrophages during carcinoma formation, especially in the M2 macrophages (Fig. 6); however, this elevation was not sustained in the later stages of the UC-CRC transition. These results indicate that TGF-β may have an essential role in the proliferation of CRC cells in the earlier stages of carcinogenesis.

The mRNA expression levels of intercellular adhesion molecule-1 (ICAM-1) were also assessed in the M1 and M2 macrophages, as this is a factor known to have a role in the transmigration of monocytes from the circulation to tissue. ICAM-1 mRNA expression levels were markedly increased in the M2 macrophages isolated from the developing carcinoma and metastatic tissues of the AOM/DSS mice, as compared with the M1 macrophages. These results indicate that M2 macrophages may have a greater effect on attracting circulating monocytes, as compared with the M1 macrophages, during the later stages of the UC-CRC transition (Fig. 6).

## Discussion

CRC is associated with a marked increase in the number of macrophages, particularly M2 macrophage polarization, which has previously been shown to correlate with metastasis and poor prognosis of the disease (11–14,26,27). The tumor microenvironment is affected by sequential changes and pathological progression, which may lead to changes in macrophage populations and macrophage-dependent inflammatory activity. Therefore, it is critically important to genetically define the expression pattern and function of M1/M2 macrophages during inflammation, and in the tumor microenvironment.

The present study used the AOM/DSS mouse model of UC-CRC carcinogenesis. The expression of macrophages markedly increased as the disease progressed from normal mucosa to inflammatory hyperplasia, dysplasia, and carcinoma, as detected by IHC. These results were concordant with previous studies (22–24), and further confirmed the sequential changes between pathological stages and macrophage expression patterns. The M1/M2 macrophage expression pattern is complex and diverse during CRC and peritoneal metastasis transitions; therefore, it remains unclear whether M1/M2 polarization alone may explain the various functional transitions during the UC-CRC process. The most substantial changes in M1/M2 macrophage expression occurred during the period between inflammatory hyperplasia and formation of carcinoma in the colonic tissue. These results indicate that inflammatory hyperplasia is a key transition event between inflammation and the development of carcinoma.

The expression pattern of colorectal M1 macrophages was similar to that of total macrophages. Notably, the expression of colorectal M2 macrophages markedly increased in the carcinoma stage, between weeks 10 and 16; however, it hardly changed during the other stages of the disease. These data indicate that the subpopulation of M2 macrophages is relatively stable during CRC development, and is involved in the early development of carcinoma, induced by a changing microenvironment. It may be hypothesized, that the changes to the expression levels of the described factors in M1/M2 macrophages, may correlate with the functional changes during the UC-CRC process.

Macrophage activation can be either pro- or anti-inflammatory in an adaptive immune response, depending on the M1/M2 phenotype (15,28,29). Macrophage-mediated inflammation has a differential effect in different stages of colon cancer and the present study demonstrated that the pro- and anti-inflammatory axis is unbalanced in colorectal M1/M2 macrophages. The present study demonstrated a loss of anti-inflammatory IL-10, during the transition from inflammation to carcinoma formation, and the coordinated promotion of pro- and anti-inflammatory cytokines in the development of carcinoma and metastasis by M2 macrophages. These results confirm that these cells have an important role in the promotion of CRC through inhibition of the pro- and anti-inflammatory response.

Previous studies have shown that macrophages in CRC are pro-inflammatory in function, and inhibit the proliferation of tumor cells through an anti-tumor type-1 inflammatory response (30). The results of the present study suggest that M1 macrophages conferred some, but not all, of the pro-inflammatory responses during carcinoma development in the UC-CRC process. Whereas, the M2 macrophages had a weak anti-inflammatory response and expressed a strong pro-tumor influence. Furthermore, the aberrant enhancement of the pro- and anti-inflammatory axis involved in the migration and invasion of carcinoma during metastasis, by both M1 and M2 macrophages, suggested that functional changes in M1 macrophages occurred during the transition of carcinoma *in situ* to metastasis with an unchanged polarization phenotype.

Notably, the expression levels of G-CSF and GM-CSF in colorectal macrophages were markedly upregulated during inflammatory hyperplasia and metastasis, respectively. These results suggest a potential reason as to why there is a marked recruitment of colorectal macrophages, during the UC-CRC process, which is concordant with flow cytometric data. Conversely, other studies have shown that macrophage infiltration is an obligatory step for tumor cell migration, invasion, and metastasis in both CRC and breast cancer (31,32,33). Previous studies have shown that CXCR4, VEGF, TGF-β, ICAM-1 are associated with various roles of macrophages during the process of carcinoma invasion and metastasis (34–35). The present study also assessed the expression levels of CXCR4, VEGF, and TGF-β, in order to define the role of M1/M2 macrophages in migration and invasion. The results of the present study indicate that a coordinated enhancement of CXCR4 and VEGF expression in colorectal macrophages, especially M2 macrophages, contributed to invasion and peritoneal metastasis of CRC. The expression levels of TGF-β were markedly elevated during carcinoma development in colorectal M2 macrophages, indicating a potential role for TGF-β at this stage of the disease, as compared with the other stages. In addition, CXCR4, VEGF, and TGF-β expression levels were much higher in colorectal M2 macrophages, as compared with the M1 macrophages isolated from the various stages of the UC-CRC process. These results suggest that M2 macrophages have a critical role in the initiation, promotion, and metastasis of CRC, which is concordant with the changes observed in inflammatory cytokine expression. ICAM-1 expression levels were also markedly upregulated in colorectal M2 macrophages, as compared with the M1 macrophages at all stages of the disease process, thus indicating that M2 macrophages may impart a stronger effect on metastasis than M1 macrophages. The ICAM-1 expression analysis also confirmed the pro-tumor role of colorectal M2 macrophages in carcinoma progression and metastasis.

The percentage of colorectal M2 macrophages was markedly increased as the pathological states progressed in the UC-CRC process. Furthermore, colorectal M2 cells not only broke the balance of the pro- and anti-inflammatory axis, but also enhanced the expression levels of CXCR4, VEGF, TGF-β, and ICAM-1, which are all known to promote CRC and metastasis. Previous studies have shown opposite results and have indicated that M1 macrophages impart some effects on CRC development similar to those of M2 macrophages (29–32). However, the pro-tumor effects were not as severe as the effects demonstrated by M2 macrophages during UC-CRC carcinogenesis in the present study. In addition, functional changes also occurred in M1 macrophages without any change to the phenotype during the development of carcinoma and metastasis, but not during the stages of inflammation, dysplasia, or carcinoma. Functional changes in these cells, particularly those associated with inflammation and migration, occurred earlier in the process than polarization, during UC-associated carcinogenesis of the colon.

## Figures and Tables

**Table I:** Primers used for quantitative polymerase chain reaction analysis.

Gene	Forward primers (5′→3′)	Reverse primers (5′→3′)
*CXCR4*	aaagctagccgtgatcctca	caccatttcaggctttggtt
*G-CSF*	ccttcacttctgccttccag	gctcaggtctaggccaagtg
*GM-CSF*	atgcctgtcacgttgaatga	ccgtagaccctgctcgaata
*ICAM-1*	agggctggcattgttctcta	cttcagaggcaggaaacagg
*IL-1β*	accccaaaagatgaagggctgctt	tgcctgcctgaagctcttgttgat
*IL-10*	ggcagagaagcatggcccagaa	ttcacctgctccactgccttgc
*IL-12*	gccggctatccagacaatta	ggccaaactgaggtggttta
*IL-6*	ccggagaggagacttcacag	cagaattgccattgcacaac
*TGF-β*	ttgcttcagctccacagaga	tggttgtagagggcaaggac
*TNF-α*	tatggctcagggtccaactc	ctccctttgcagaactcagg
*VEGF*	tgatctgctccctccctcta	aatgctttctccgctctgaa
*GAPDH*	gggtgaggccggtgctgagtatg	ggcagaaggggcggagatgatg
